# Subtype Classification of Iranian HIV-1 Sequences Registered in the HIV Databases, 2006-2013

**DOI:** 10.1371/journal.pone.0105098

**Published:** 2014-09-04

**Authors:** Kazem Baesi, Samaneh Moallemi, Molood Farrokhi, Seyed Ahmad Seyed Alinaghi, Hong–Ha M. Truong

**Affiliations:** 1 Iranian Research Center for HIV/AIDS, Iranian Institute for Reduction of High Risk Behaviors, Tehran University of Medical Sciences, Tehran, Iran; 2 University of California, San Francisco, CA, United States of America; 3 Gladstone Institute of Virology and Immunology, San Francisco, CA, United States of America; University of Athens, Medical School, Greece

## Abstract

**Background:**

The rate of human immunodeficiency virus type 1 (HIV-1) infection in Iran has increased dramatically in the past few years. While the earliest cases were among hemophiliacs, injection drug users (IDUs) fuel the current epidemic. Previous molecular epidemiological analysis found that subtype A was most common among IDUs but more recent studies suggest CRF_35AD may be more prevalent now. To gain a better understanding of the molecular epidemiology of HIV-1 infection in Iran, we analyzed all Iranian HIV sequence data from the Los Alamos National Laboratory.

**Methods:**

All Iranian HIV sequences from subtyping studies with *pol*, *gag*, *env* and full-length HIV-1 genome sequences registered in the HIV databases (www.hiv.lanl.gov) between 2006 and 2013 were downloaded. Phylogenetic trees of each region were constructed using Neighbor-Joining (NJ) and Maximum Parsimony methods.

**Results:**

A total of 475 HIV sequences were analyzed. Overall, 78% of sequences were CRF_35AD. By gene region, CRF_35AD comprised 83% of HIV-1 *pol*, 62% of *env*, 78% of *gag*, and 90% of full-length genome sequences analyzed. There were 240 sequences re-categorized as CRF_AD. The proportion of CRF_35AD sequences categorized by the present study is nearly double the proportion of what had been reported.

**Conclusions:**

Phylogenetic analysis indicates HIV-1 subtype CRF_35AD is the predominant circulating strain in Iran. This result differed from previous studies that reported subtype A as most prevalent in HIV- infected patients but confirmed other studies which reported CRF_35AD as predominant among IDUs. The observed epidemiological connection between HIV strains circulating in Iran and Afghanistan may be due to drug trafficking and/or immigration between the two countries. This finding suggests the possible origins and transmission dynamics of HIV/AIDS within Iran and provides useful information for designing control and intervention strategies.

## Introduction

Important characteristics that contribute to the worldwide spread of HIV are its enormous genetic variability and rapid evolution, which makes the virus highly adaptable to selection pressures of new hosts. The high error rate of the reverse transcriptase which lacks a proofreading mechanism, high rates of virus production in vivo, persistent nature of infection and selective immune pressure are factors responsible for the high genetic variation of HIV. [1] The presence of viral RNA as a dimer and co-infection of a cell with more than one viral genotype also results in recombination and mixed genotypes. Together, this presents a complex picture of genetic variation of HIV-1 virus. [2]

The genetic variability of HIV affects pathogenesis, immune response and escape, vaccine development, transmission, disease progression, drug resistance and treatment response. [3], [4] Therefore, molecular epidemiology studies are extremely important to characterize the HIV-1 subtype distribution in a specific population/region which may significantly influence diagnostic and therapeutic strategies. [5]

HIV-1 has four distinct genetic groups: M, N, O, and P. When the genetic groups are presented on a phylogenetic tree, strains within group M form well-defined clusters. Nine distinct subtypes (A–D, F–H, J, and K) have been identified, along with 61 circulating recombinant forms (CRFs) which are inter-subtype recombinant strains. [6]

The rate of human immunodeficiency virus type1 (HIV-1) infection in Iran has increased dramatically in the past few years. While the earliest cases were among hemophiliacs, injection drug users (IDUs) fuel the current epidemic. According to the CDC, a total of 26,556 PLWH had been identified in Iran through June 2013.[7]–[9] The HIV transmission routes in all the cases registered since 1986, in order of magnitude, are sharing injection equipment among IDUs (68.4%), sexual intercourse (12.3%), blood transfusion (0.9%), and mother-to-child transmission (1.2%); the route of transmission for the remaining 17.2% is unknown. [7]

In Iran, previous molecular epidemiological analysis of HIV-1 *gag* and *env* gene segments found that the predominant strain circulating among IDUs was subtype A which was related to African Ugandan/Kenyan sub-Saharan isolates.[10]–[13] More recent studies of *pol*, *gag* and *env* gene segments reported that the predominant strain was CRF_35AD.[6], [14]–[16] To gain a better understanding of the molecular epidemiology of HIV-1 infection in Iran, we analyzed all Iranian HIV sequence data from the Los Alamos National Laboratory.

## Methods

A secondary analysis was performed using all Iranian HIV sequences from subtyping studies with *pol*, *gag*, *env* and full-length HIV-1 genome sequences registered in the HIV databases at the Los Alamos National Laboratory (www.hiv.lanl.gov) between 2006 and 2013. The sequences were downloaded along with reference nucleotide sequences for those regions [accession numbers: AB703607-AB703616, AY693842-AY693971, DQ077824-DQ077851, DQ077854-DQ077871, DQ115645-DQ115707, DQ149128-DQ149133, DQ788541-DQ788560, EU881931, FJ178375, FJ178376, FJ392730-FJ392755, FJ790242, FJ807629, FJ807630, GQ243705-GQ243708, GQ273945-GQ273960, GQ274861-GQ274878, GQ853425-GQ853428, GU724804-GU724845, HQ233645, HQ735066, HQ735068, KF029508-KF029592, EF158040- EF158043, GQ477442-GQ477451, DQ676872, AB253421, AB253429, AF286238, GU201516, AF286237, K03455, AY523387, AY173951, AY331295, U52953, U46016, AF067155, AY772699, K03454, AY371157, AY253311, U88824, AF077336, AF005494, AF075703, AJ249238, AY371158, AJ249236, AJ249237, AF377956, AF084936, AF061641, U88826, AY612637, AF190127, AF190128, AF005496, FJ711703, EF614151, GU237072, AF082394, AJ249239, GQ477441, GU564221, U54771, AY271690, AB485636, L39106, AF193276, AF049337, AF119819, AF119820, AF076998, AF193253, AY227107, AF064699, AY535659, AB286851, EF368372, EF368370, AF286230, HM067748, AY008715, AJ866553, AY093605, AY093603, AY093607, AF289548-AF289550, AF492623, AF492624, AF408629, AF408630, AF385936, DQ845388, AF450096, AF450097, DQ354120, AF516184, AY945736, AF286239, EU581825, EU581827, EU581828, AF377959, AY586541, AY894993, AY588971, AY588970, AY894994, AY586545, AY945737, AF457051, AF457072, AY371159, GQ229529, AY900571, AY900572, AY900574, AY900575, AJ670526, EU693240, EU697906, EU697908, FM877780, FM877782, FM877777, AJ404325, AM851091, DQ085872, DQ085873, DQ085874, DQ085876, AY771590, DQ085871, EF091932, AY727526, AY727527, AY535660, AB547464, DQ366659, DQ366662, EF165541, EF158043, EF158040, EF158041, EF087995, EF087994, EF116594, AF377957, FJ213781, FJ213782, FJ213780, EU735534, EU735536, EU735535, EU735538, EU735540, EU735339, EU170155, EU697904, EU697907, EU697909, FJ358521, FN392874, FN392876, FN392877, DQ358801, DQ358802, HM026456, GQ372987, FJ670529, HQ385477, HQ385479, HQ385478, L20587, L20571, AY169812, AJ302647, AY532635, AJ006022, AJ271370, HQ179987, GU111555, U42720, DQ373066, AF103818]. *Pol*, *gag* and *env* genes have been shown to be reliable regions for HIV-1 subtyping. [8], [17]–[19]

Nucleotide sequences from each gene were aligned with the reference sequences using CLUSTAL W software. Phylogenetic trees were constructed using Neighbor-Joining (NJ) and Maximum Parsimony methods (1000 times bootstrap replicates) with Molecular Evolutionary Genetics Analysis (MEGA) software version 5. [20] The Kimura 2-parameter model was used with a transition/transversion ratio of 1.5 and statistical support of the tree structures was obtained with 1000 bootstrap replicates. Significance was based on bootstrap values of >70%. [20]

To confirm the results obtained using MEGA5, sequences were re-analyzed using REGA. To improve the accuracy of the characterization of recombinant forms, Maximum Likelihood and NJ trees were re-constructed using RDP v.4.35 software. Results from all the different analyses were compared to determine the final subtype characterizations.

## Results

A total of 475 Iranian HIV-1 sequences were analyzed in this study, of which 174 sequences were for *pol* genes, 161 sequences for *env* genes, 130 sequences for *gag* genes, and 10 sequences for full-length genomes. Overall, CRF_35AD was the predominant subtype representing 78% of sequences. By region, CRF_35AD comprised 83% of HIV-1 *pol* sequences, 62% of *env* sequences, 78% of *gag* sequences, and 90% of full-length genome sequences analyzed.

Table 1 presents the distribution of subtypes in the present study. There were 240 sequences re-categorized as CRF_AD and 2 sequences re-categorized as CRF_29BF. The proportion of CRF_35AD sequences categorized by the current study is nearly double the proportion of what had been reported in the HIV databases. Of the HIV-1 *pol* gene sequences analyzed, 69 A1 sequences (39.6%) in the Baesi and Hamkar studies and 55 CRF_AD sequences (31.6%) in the Soheili and Hamkar studies were re-categorized as CRF_35AD in present study and 2 sequences (1.1%) which had been reported as subtype B in the Hamkar study were re-categorized as subtype CRF_29BF. [10], [14], [16] Of the HIV-1 *env* gene sequences, 15 sequences (9.3%) which had been reported as subtype A1 in the Bahmani and Khosravi studies were re-classified as subtype CRF_35AD. [20] Of the HIV-1 *gag* gene sequences, 101 A1 sequences (77.7%) in the Naderi and Sarami studies were re-categorized as subtype CRF35_AD. [11], [12]

**Table 1:** HIV-1 subtype classification by gene region of HIV-1 Iranian sequences from the HIV databases (www.hiv.lanl.gov).

	HIV-1 Genes			
**Subtype** Number (%)	***pol***	***env***	***gag***	**Full-length genome**
	CRF_35AD 144 (82.8%)	CRF_35AD 100 (62.1%)	CRF_35AD 101 (77.7%)	CRF_35AD 9 (90.0%)
	CRF_AE 2 (1.1%)	CRF_AE 1 (0.6%)	–	AE 1 (10.0%)
	–	–	A1 2 (1.5%)	–
	B 25 (14.4%)	B 54 (33.5%)	B 27 (20.7%)	–
	CRF_29BF 2 (1.1%)	–	–	–
	C 1 (0.6%)	C 6 (3.7%)	–	–
**Total**	174	161	130	10

**Figure 1:**
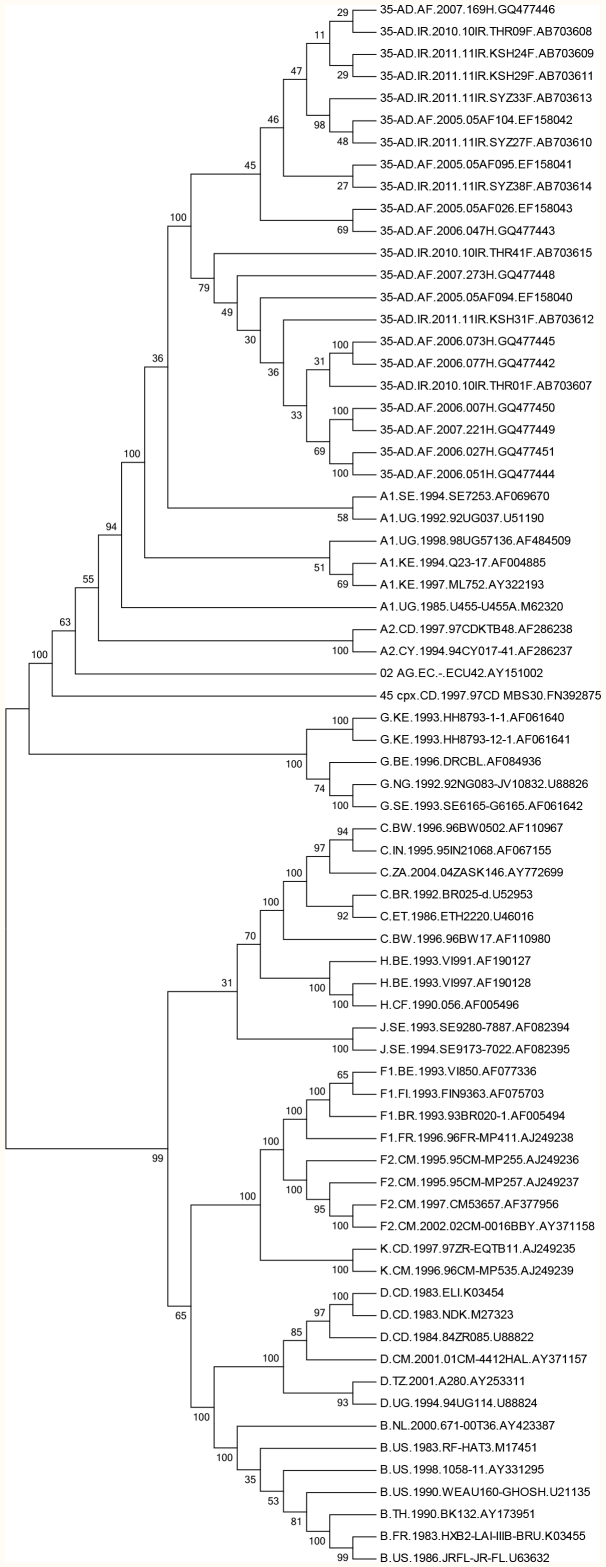
Un-rooted phylogenetic tree of CRF_AD whole genome sequences from Iran and Afghanistan, constructed using Kimura 2-parameter matrices and Neighbor-Joining method.

A phylogenetic tree of HIV whole genome sequences from Iran and Afghanistan is shown in Figure 1. The 13 Afghani isolates were from the mid- to late 2000’s whereas the 9 Iranian isolates were from the early 2010’s. In the three pairs that were comprised of an isolate from Afghanistan clustering with an isolate from Iran, the support values were low.

## Discussion

In this phylogenetic analysis of sequences in the Iranian population, HIV-1 subtype CRF_35AD was found to be the dominant circulating strain. The result of this analysis differed from previous studies which reported subtype A as the most prevalent in HIV- infected patients in Iran but confirmed the results of other studies which reported the predominance of HIV-1 CRF_35AD among Iranian IDUs. The phylogenetic analysis also identified 2 cases of CRF_29BF. The observed differences between our findings and those of previous studies may be due to the unavailability of HIV reference sequences for certain subtypes, e.g., CRF_29BF, in the HIV databases in previous years or alignments performed using reference sequences that were not representative of all subtypes in Iran.

The identification of CRF_35AD and CRF_29BF strains circulating in Iran are likely the result of the importation of these strains from other countries. The observed epidemiological connection between HIV strains circulating in Iran and Afghanistan may be due to drug trafficking and/or immigration between these two countries. Iran is a major route for drug trafficking between Afghanistan and Europe. In addition, Iran has received a large number of Afghan refugees. Since CRF_35AD is also the dominant strain among Afghan IDUs, it is possible that the observed expansion of CRF_35AD is due in part to Afghan IDUs who immigrated to Iran. [6], [21], [22] CRF_29 strains in Iran may have originated from South America where this subtype is more commonly found.

Our findings suggest the possible origins and transmission dynamics of HIV/AIDS within Iran. Knowing the distribution of HIV variants alongside the corresponding epidemiologic factors will help assess the implications of any differences in transmissibility. The public health implications of such findings, including prevention and treatment strategies, are of special interest. According to the latest report from CDC, injection drug use remains the primary transmission route of HIV infection in the country. Therefore, current harm reduction programs for IDUs in Iran need to be strengthened to prevent further HIV transmission among IDUs and to other populations. This molecular epidemiological information will also be extremely relevant for guiding the development and implementation of diagnostic as well as preventive and therapeutic approaches in Iran.
